# A multidimensional approach to inform family planning needs, preferences and behaviours amongst women in South Africa through body mapping

**DOI:** 10.1186/s12978-019-0830-6

**Published:** 2019-11-06

**Authors:** Jane Harries, Deborah Constant, Vanessa Wright, Chelsea Morroni, Alex Müller, Christopher J. Colvin

**Affiliations:** 1 Women’s Health Research Unit, School of Public Health and Family Medicine, University of Cape Town, Cape Town, South Africa; 2 Department of International Public Health, Liverpool School of Tropical Medicine, Liverpool, UK; 3 Gender Health and Justice Research Unit, Faculty of Health Sciences, University of Cape Town, Cape Town, South Africa; 4 Division of Social and Behavioural Sciences, School of Public Health and Family Medicine, University of Cape Town, Cape Town, South Africa

**Keywords:** Contraception, Family planning, Unintended pregnancy, South Africa, Body mapping, Participatory research, Sensory experiences, Person-Centred counseling

## Abstract

**Background:**

In recent decades there have been great improvements in the reproductive health of women in low- and middle-income countries and increases in the use of modern contraceptive methods. Nonetheless, many women are not able to access information, contraceptive technologies and services that could facilitate preventing unintended pregnancies and planning the number and timing of desired pregnancies.

In South Africa, the contraceptive prevalence rate is 64.6%. However, this relatively high contraceptive prevalence rate masks problems with quality contraceptive service delivery, equitable access, and women’s ability to correctly and consistently, use contraceptive methods of their choice. This study set out to understand the specific family planning and contraceptive needs and behaviours of women of reproductive age in South Africa, through a lived experience, multisensory approach.

**Methods:**

Participatory qualitative research methods were used including body mapping workshops amongst reproductive aged women recruited from urban and peri urban areas in the Western Cape South Africa. Data including body map images were analysed using a thematic analysis approach.

**Results:**

Women had limited biomedical knowledge of the female reproductive anatomy, conception, fertility and how contraceptives worked, compounded by a lack of contraceptive counseling and support from health care providers. Women’s preferences for different contraceptive methods were not based on a single, sensory or experiential factor. Rather, they were made up of a composite of sensory, physical, social and emotional experiences underscored by potential for threats to bodily harm.

**Conclusions:**

This study highlighted the need to address communication and knowledge gaps around the female reproductive anatomy, different contraceptive methods and how contraception works to prevent a pregnancy. Women, including younger women, identified sexual and reproductive health knowledge gaps themselves and identified these gaps as important factors that influenced uptake and effective contraceptive use. These knowledge gaps were overwhelmingly linked to poor or absent communication and counseling provided by health care providers. Body mapping techniques could be used in education and communication strategies around sexual and reproductive health programmes in diverse settings.

## Plain English summary

This study set out to understand the family planning and contraceptive needs and behaviours of women of reproductive age in South Africa. This was achieved through exploring women’s biomedical knowledge of the reproductive system and contraception including the physical and sensory experiences of contraceptives and how this influenced contraceptive decision making. Body mapping workshops and group discussions with fifty-seven women of reproductive age (18–49 years) were used to explore women’s physical and sensory experiences of contraceptive methods. Body mapping is the process of creating life-sized figures using drawing or painting to visually represent aspects of peoples’ lives, their bodies and the world they live in. Body maps can be life-size, smaller in size, or using smaller, pre- drawn outlines or templates of bodies.

This study highlighted the need to address communication and knowledge gaps around the female reproductive system, different contraceptive methods and how contraception works to prevent a pregnancy. Women’s poor levels of knowledge of their reproductive system and contraception and family planning were linked to poor or absent communication and counseling provided by the health care services. Body mapping methods could be used in education and communication strategies around sexual and reproductive health programmes in South Africa and elsewhere.

## Background

In recent decades there have been great improvements in the reproductive health of women and men in low and middle income countries, and increases in the use of modern contraceptive methods [1]. Nonetheless, many women, couples and adolescents do not or are not able to access information, contraceptive technologies and services that could facilitate preventing unintended pregnancies and planning the number and timing of desired pregnancies. The optimal use of modern contraceptive methods can help prevent unintended pregnancies and induced abortions in low- and middle-income countries, reduce maternal and infant mortality and improve social, economic and educational outcomes for women and their families [1–4].

In South Africa, the contraceptive prevalence rate for all women of reproductive age (15–49 years) who are using a modern contraceptive method is 64.6%. However, this relatively high contraceptive prevalence rate masks problems with service delivery; equitable access, and correct, consistent, and continuous use of contraception especially among certain groups such as young or rural women [5, 6].

Further research is critical to understand the barriers of long acting reversible contraceptive use, particularly hormonal implants and intrauterine devices amongst South African women.

This study, which formed part of a Bill & Melinda Gates Grand Challenges Explorations grant, set out to understand the specific family planning and contraceptive needs and behaviours of women of reproductive age in South Africa, through a lived experience, multisensory approach. This approach explored women’s day-to-day behaviours, knowledge of their reproductive system, and interactions with modern contraceptive methods from multiple perspectives, including physical, tactile and sensory experiences. Research on women’s perception about modern contraceptive methods has typically focused on side effects and the limited contraceptive choices that affect women’s decision-making and use. Less is known about how women’s physical and sensory experiences of contraceptive technologies and their daily lived experiences of contraceptive use affect their perceptions and decision-making.

## Methods

### Study design

Body mapping, an innovative participatory methodology, was considered the most suitable method to explore women’s embodied experiences and understanding of their reproductive physiology and contraception. Body mapping is the process of creating life-sized figures or smaller scale pre-drawn outlines using drawing, painting or other visual art techniques to visually represent aspects of peoples’ lives, their bodies and the world they live in [7]. This particular genre of body mapping originated in South Africa as a visual and arts-based therapeutic activity for women living with HIV/AIDS [8–11]. The use of body mapping to describe the sensory and lived experiences of contraceptive use, in particular, is novel in the sexual and reproductive health field. We wanted to adapt and expand this participatory research method to issues of reproduction, contraception and family planning. We adapted the body mapping method to explore and visually represent the intersections of women’s knowledge of their reproductive anatomy and physiology, menstruation, conception, fertility, contraception and family planning. Body mapping workshops offered participants the opportunity for non-verbal expressions of body experiences and were helpful for those not accustomed to verbalising sensitive or private experiences.

### Research setting and study population

Data was collected between February 2017 – March 2018 in four urban and peri-urban areas in the Western Cape Province, South Africa. Study participants were recruited through the assistance of community outreach workers from communities in close proximity to our research sites. Accessing participants outside of the health care system enabled us to reach women who are less likely to regularly attend health care facilities and who are often overlooked and underserved with respect to family planning services. Populations of interest included married and unmarried women of reproductive age (18–49), nulliparous and parous women, current users, past users and non-users of modern contraception, and married and unmarried men. Research undertaken with men will be reported elsewhere.

Key informants from non-governmental organisations and a community outreach worker working amongst women assisted with study recruitment. We employed convenience sampling based on age > 18 years and willingness to participate in 1–2-day body mapping workshops. In total we conducted six body mapping workshops of one to 2 days duration amongst women.

### Data collection

**Fig. 1:**
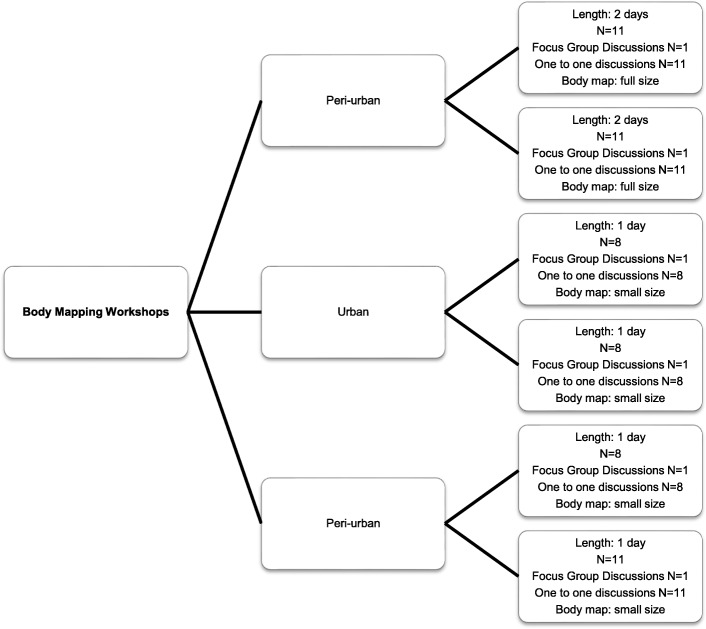
Body mapping workshops

Participatory qualitative research methods were used, and data were collected through three data collection methods; i) 1–2 day body mapping workshops with women, followed by ii) private one-on-one and then iii) focus group discussions with all women at the end of the body mapping workshop day. (See Fig. 1) In total, 57 women and 28 men participated in the study. In this article, we report only on the body mapping and discussions with women. Detailed analysis of the men’s focus group discussions will be presented elsewhere. Research assistants and study team members trained in qualitative research methods (including body mapping methods) and with a background in sexual and reproductive health introduced the study to participants who had been invited to participate by community outreach workers.

Body mapping workshops were conducted by female members of the research team who were local to the Western Cape of South Africa, included the first author, second author and third author, the latter was a Canadian primary health care nurse practitioner residing in South Africa and had experience in body mapping work in other settings. Informed consent was obtained from all study participants prior to commencement of the workshops. Focus group discussions were part of the body mapping workshops and usually occurred towards the end of the day. Socio-demographic information including age, employment, educational level, marital status, past and current contraceptive use and knowledge, number of children and future fertility intentions was collected prior to commencement of body mapping workshops and focus group discussions. Over the 2 days, involvement in art making and group discussions facilitated free discussion on a sensitive topic, which was felt to be particularly relevant when discussing sexual and reproductive health enabled by the participatory nature of the body mapping workshops. Body mapping workshops were conducted in local community halls. All study participants were reimbursed ZAR150 per day. Workshops were conducted in both English and Afrikaans depending on study participants’ language preference. The first author and research assistant were both conversant in English and Afrikaans ensuring that meanings were not lost in translation. The research assistant (not an author) had prior experience working in these communities and verified words or phrases (local vernacular) that we were not familiar with.

**Fig. 2:**
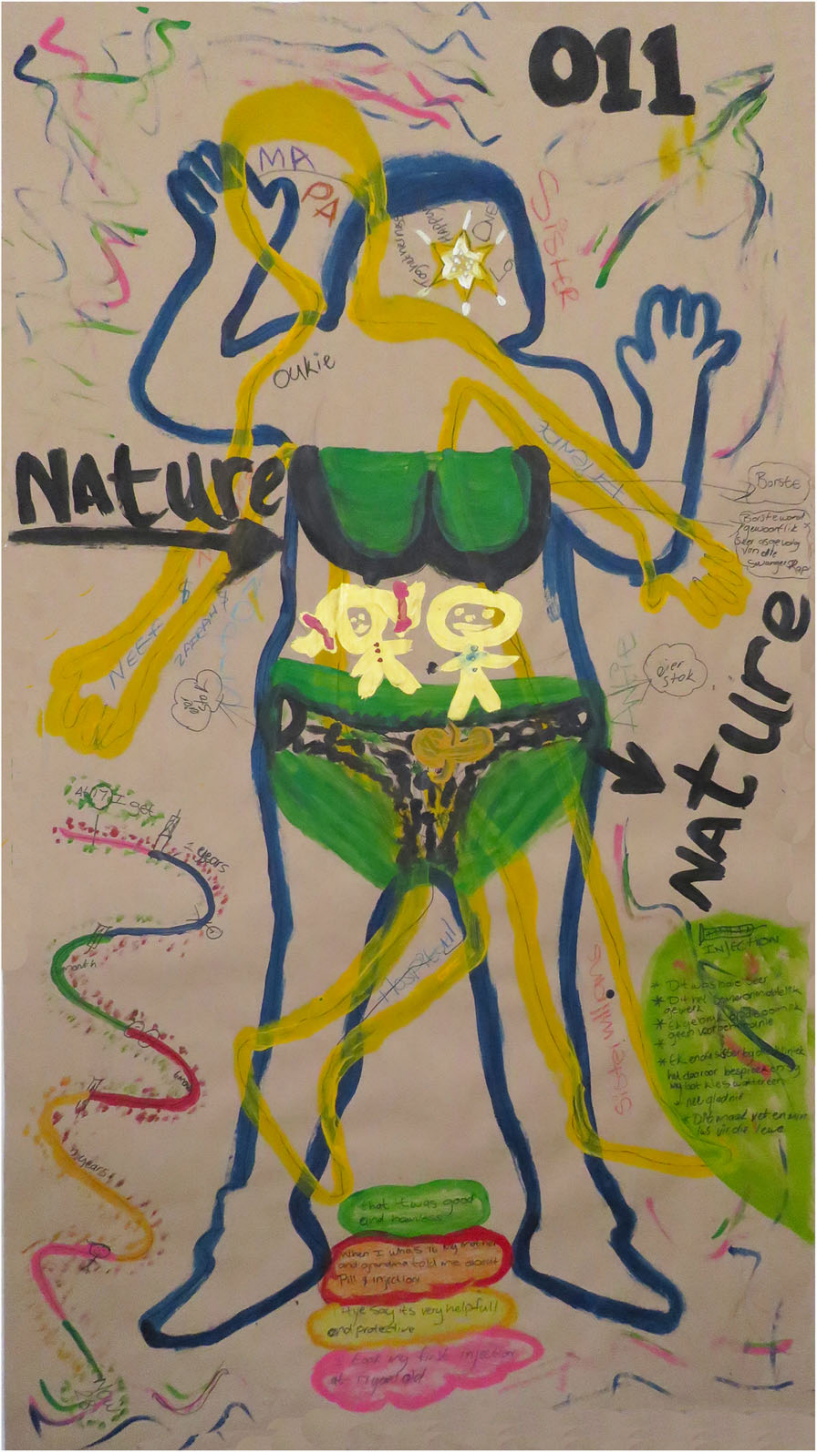
Body map and contraceptive timeline

**Fig. 3:**
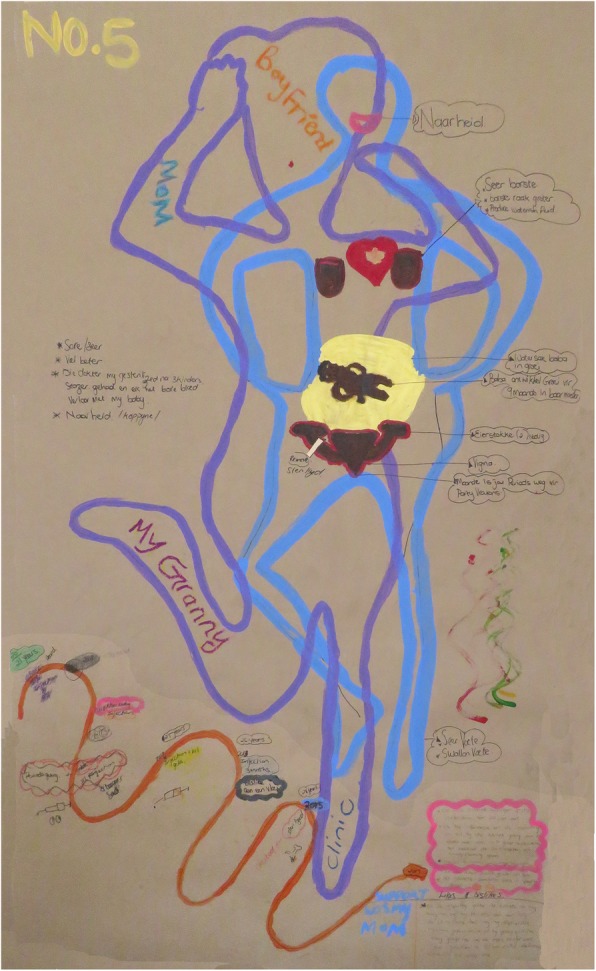
Body map and contraceptive timeline

Body mapping workshops were undertaken by an experienced female body mapping facilitator and consisted of 7–12 study participants per workshop. In total we conducted 6 body mapping workshops of 1–2 days duration using both small (self drawn) and life size body maps. Each woman created their own body maps during different stages of the body mapping workshop. Through art making activities including the symbolic use of colour, key areas explored over a 2-day programme included levels of knowledge and experiences around the female reproductive anatomy, conception, fertility and contraception including different contraceptive methods. In addition, individual and focus group discussions were conducted with women during body mapping sessions to explore and examine in-depth visual connections and meanings around their individual body maps. This included depicting on the body maps how they felt within their body and in relation to their community, family, partners and friends, as well as their interaction with health care facilities. Key areas explored during the body mapping workshops and discussions included knowledge and understanding of the female sexual and reproductive health system, and knowledge and understanding of methods to prevent an unintended pregnancy with a specific focus on contraceptive choice, uptake and usage. The latter was displayed through visual representation on a contraceptive timeline. We asked participants to create a contraceptive timeline using drawing, painting and writing to show an overview of their contraceptive history. On the same timeline we asked them to mark the births of any children and the year in which they were born. At the end of the timeline we asked participants to identify their current form of contraception and how it made them feel within their body. Figures 2 and 3 Body map and contraceptive timeline.

During the body mapping workshops and discussions, women requested more information around contraception to understand potential side effects as well as to better assess information obtained through social networks and from health care providers. We decided to provide an interactive educational session at the end of the workshops answering questions and providing information on reproduction, ovulation, fertility, the menstrual cycle, sexually transmitted infections and different contraceptive methods and how they work. We were assisted in this process by a member of the research team, a female primary health care nurse practitioner. The objective was to increase biomedical knowledge of female reproductive anatomy through interactive group participation.. The session was facilitated through an interactive question and answer teaching style. The educational session was purposefully grounded in human anatomy and the female reproductive life cycle to allow participants to explore their own understanding of sexual health, relationships, and contraceptive choices individually or within the group. The women reported they benefited from this group education and participation.

### Ethical considerations

Ethical approval was obtained from the Human Research Ethics Committee at the University of Cape Town (HREC REF 092/2017). All study participants provided written informed consent. Permission was also obtained to digitally record all interviews and photograph participants alongside their body maps. Participants were engaged in the body mapping process and requested to be photographed alongside their body maps. Women could opt to not be photographed alongside their body maps, Confidentiality and anonymity were ensured. Participants were assured that in all forms of dissemination, including publications and dissemination meetings, participants would not be identified by name or any other identifier including research sites. Written permission as part of the consent process was obtained to reproduce body maps and other images including photographs. All data were closely controlled by the principal investigator and stored in locked cabinets and digital copies stored on a password protected computer. Digital recordings were erased once they had been cross checked after data transcription. Women were offered their large body maps at the end of the body mapping workshops. Most women did not have the space to store their life-sized body maps and a printed photograph of their body map was provided to all women. All body maps have been stored in a secure space at the study offices with restricted access.

### Data analysis

Data analysis included both verbal and visual interpretation of data by the study team. All focus group and individual one on one discussions were digitally recorded and transcribed by an independent transcriber. Two members of the research team verified all transcripts for accuracy. Body maps and contraceptive timeline images were visually examined and all text and notations on the maps and timelines were manually recorded for each study participant. The research team sat with each participant and discussed the different drawings and text on their body map. Participants also presented their final body maps to the group and discussed what their images meant. We recorded these sessions and took notes. Discussions accompanying the body mapping workshops were also included in analysis. All data was broadly coded (manually) following the key issues explored during the workshops and discussions.

Critical examination of participants’ visual and oral narratives focused on several key themes. Women’s contraceptive needs, behaviors and experiences were explored from an ecological perspective, which emphasized both individual and contextual factors, and the interdependent and dynamic interrelations between the two. Data were further analyzed using a thematic analysis approach, in which main themes and categories were identified and analyzed within and across all forms of data. The analysis was essentially data driven, and initial categories for analyzing data were drawn from the key areas explored during the body mapping workshops. We triangulated findings across the different sites and methods and clarified interpretations with research team members and with relevant literature [7, 10, 12, 13].

## Results

### Participant characteristics

Table 1 presents the socio-demographic characteristics of the study participants. The mean age for women (*n* = 57) was 27.2 years (range 18–45), 12% were married, 91% unemployed, 42% had completed high school, 28% were not currently using a contraceptive method, and of those women using a contraceptive method, 47% used the injectable contraceptive. Almost 80% of women had one or more children and over half of the women (51%) did not plan to have more children.

**Table 1:** Sociodemographic characteristics of women participants (*n* = 57)

Age (years)		
18–20	7	12%
21–30	36	63%
31–50	14	25%
Marital status		
Single	47	82%
Married	7	12%
Other (divorced, engaged)	3	5%
Home language		
English	1	2%
Xhosa	21	37%
Afrikaans	35	61%
Highest level of education completed		
< =Grade 11	33	58%
Grade 12	21	37%
Post-secondary	3	5%
Employment status		
Employed	5	9%
Unemployed	52	91%
Currently using contraception	41	72%
Current contraceptive method (*n* = 41)		
No method	16	28%
Female sterilization	3	5%
IUD	1	2%
Implant	5	9%
Injection	27	47%
Oral contraceptive pill	2	4%
Male or Female condom	3	5%
Prior contraceptive method		
Never used	9	16%
IUD	1	2%
Implant	3	5%
Injection	36	63%
Oral contraceptive pill	5	9%
Male or Female condom	3	5%
Current number of children		
0	12	21%
1	22	39%
2	15	26%
3–6	8	14%
Number of children desired in the future (*n* = 56)		
0	29	52%
1	12	21%
2–4	15	27%

Key areas explored during the body mapping workshops and discussions included knowledge and understandings of the sexual and reproductive health system including ways to prevent an unintended pregnancy, with a specific focus on contraceptive characteristics, uptake and usage, the latter through visual representation on a contraceptive timeline. The influence of social networks, including partner relations, were explored, including women’s experiences with family planning services.

### Knowledge of reproductive system

Overall, women had limited biomedical knowledge of the female reproductive anatomy, conception, fertility and how contraceptives worked, which seemed to be compounded by a lack of contraceptive counseling and support from health care providers. Body map images foregrounded existing local perceptions and reproductive health knowledge. Most women located “where the baby develops” outside of the uterus, in the abdomen, and often relied on the local vernacular when labelling the reproductive organs using words such as *muis* (mouse) and *cupcake* for the vagina.

Information around contraceptive methods obtained from community and social networks influenced contraceptive uptake and use. For example, one group of women highlighted the belief that the progestogen-only injectable contraceptive Depo-Provera widely used in South Africa, could cause cancer:

“*We heard a lot of stories that Depo is not good for the body, so we didn’t want it … because it actually has different effects and I heard that someone also got cancer from it*”.

Women also spoke about fears around infertility, which they attributed to changing bleeding patterns. This fear of infertility and harmful health effects of bleeding changes was prevalent whether women were taking a progestin-only contraceptive method or not, and the connection that progestin-only contraception may alter bleeding patterns more significantly than some of the other methods available such as the combined oral pill or the copper intrauterine device was not made by participants.

### Contraceptive characteristics

Women’s preferences for different contraceptive methods were not based on a single, sensory or experiential factor. Rather, they were made up of a composite of sensory, physical, social and emotional experiences underscored by potential for threats to bodily harm. Decision-making around contraceptive uptake, including consistent and correct use, were influenced by wider community networks including family, partners and friends, underscored by broader gender dynamics and health system factors. Women’s decision making in relation to contraceptive use were thus a result of the interplay between multifaceted influences at individual and structural level. This supported our initial understandings that women’s decision making does not occur in a vacuum but are rather located within their wider social context.

A group of women recounted how they initially heard about contraception and how this influenced uptake and use. Although the local clinic and school were sometimes mentioned as sources of information, most women were told about family planning initially by a family member and sometimes a close friend. Many women expressed that it was important that contraception was discussed within a safe relationship. A woman recounted how her mother was the first one to inform her about family planning.

*“Mom told me about family planning… I was very calm at that stage because I had a very open relationship with my mother and could talk about anything and felt my mom trusted me”*

How the contraceptive felt, smelt, looked and tasted (particularly flavoured male condoms) all played a role in women’s decision making around method use. This was noted particularly with the sub-dermal contraceptive implant, where being able to see the implant, once placed in a woman’s arm, was both a safety risk to be violently removed by gangsters and intrusion on personal privacy. Visibility and discretion played a key role. Contraception was viewed by participants as a practice hidden from partners and community members. Contraceptive visibility could jeopardize physical safety. The potential visibility of the implant was a marker that one was on contraception and hence sexually active. This was an issue in a close-knit community where discretion was important. A younger woman attending a body mapping workshop explained:

“*On a thin person, one can see the line [implant]…It’s like a scar or foreign object – I was shocked when I saw my friend was on the implant and she hadn’t told me*”.

Furthermore, some women wanted to conceal contraceptive use from boyfriends or sexual partners. A woman suggested another less visible location in the body for placement of the implant such as the abdomen.

“*Because maybe if your boyfriend wants a child … I’ve got two children, I don’t want more children. Now maybe I take another man and he wants a child, then I’m going to tell him, no, I’m off the implant. But now he sees that there’s something that shows I’m still using the implant that won’t be a good thing*”.

Narratives involving implants being violently removed from women’s arms by gang members to be smoked as a recreational drug were also reported by women in both rural and peri urban sites and deterred some women from receiving or retaining the implant.

“*They see that you have the implant, they [gangsters] cut your arm and then take it [implant] for smoking... so we are scared of it”*.

This has been supported by research in other communities with high levels of violence and a very real threat within the context of high levels of gang violence in the Western Cape [personal communications Anna Brown 2017].

### Ease of use and duration

Women related to contraceptives in different ways: to the type of contraceptive (short or long acting, injectable, oral, contraceptive, implant and intrauterine device (IUD), physical and sensory experiences including side effects, and broader logistical and structural issues. Decisions around short or long acting contraception were often linked to women’s everyday lives. The need to take the pill daily was perceived as a challenge by some, which could result in default and pregnancy. Longer acting methods were typically preferred, as this decreased clinic visits including long wait times and reliance on memory. A woman explained:

*At the age of 29, I started to use a loop [IUD] because I want to be free… Now I am 33 and free and happy”.*

In contrast, for some women, visiting the clinic every 3 months provided certainty and deflected the responsibility of remembering to take the pill daily to the health care system.

“*You drink the pill at home … but if you know you have to be at the clinic after 3 months, you know you’re going to get it. … I’m at home and then you just forget the pill … then you’re pregnant, that’s why I use the 3 months injection”.*

The complexity of short and long acting methods also emerged in discussions around “ideal” contraceptives, where some women advocated for a method to be administered on a yearly basis to avoid visiting a health care facility; yet other women felt it was important to receive regular information and “health *check-ups*”, thus balancing the need for autonomy against preventive health care.

### Side effects

Physical side effects associated with certain contraceptive methods played a significant role in uptake and consistent use. Side effects discussed by participants included weight loss or weight gain, menstrual cycle changes, painful periods, bloating, nausea, dizziness, moodiness, headaches, loss of libido, body aches, changes in appetite and tiredness. Changes in body weight and menstrual cycle changes seemed of greatest concern to women, and were often the reason why women changed their contraceptive method, or discontinued it. Amenorrhea was similarly a concern as a group of women stated:

“*Blood must come out! –you get pain because the blood doesn’t come out”.*

Whilst sensory and physical experiences related to contraceptive characteristics influenced contraceptive uptake and continuation, women also alluded to more personal motivations as influencing contraceptive use especially continued use. A group of women explained that being self-motivated, independent and actively seeking out sexual and reproductive health information was important whereas women who “don’t ask questions, don’t get informed” and could result in unintended pregnancies*.*

### Social networks

Women’s individual contexts and wider social networks had a significant influence on contraceptive uptake. This was explained by a group of women highlighting the need for person-centred counseling and recognition of wider social influences:

“*You can get the knowledge, but everybody doesn’t understand it and take it the same way, someone else will have a whole other opinion on contraception … That’s why we have different opinions and we choose different kinds of contraceptives”*.

Relationship dynamics influenced contraceptive use and when women entered new relationships, or their relationship changed, contraception methods often changed as well. The contraceptive timelines created in the body mapping workshops suggested sporadic and intermittent contraceptive use, usually initiated after the birth of their first child at a young age. A younger woman explained:

“*I was not dating so I decided to leave the injection because I had no boyfriend. I was happy about not being on the injection”.*

Similarly, another woman concurred*:*

*“At the moment I am using nothing because I have no boyfriend and I am not having sex”.*

### Health systems factors

Contraceptive preferences and use were influenced by the physical and sensory attributes of contraceptive technologies including ease of use and duration discussed above. However, less than optimal contraceptive and family planning services played a key role in women’s decision making around contraceptive initiation, uptake and consistent use. In the South African health system, women typically access their contraceptives and information about family planning through free public sector clinics. Poor service delivery including long wait times, limited information and choice and judgmental staff attitudes were all described as hindering access to contraceptive services. The need for comprehensive counseling and inclusion in contraceptive decision making in both the postpartum and family planning setting were highlighted by women in all research sites. A woman in a group discussion explained:

*“I think there should be a system where they give the list of these things [contraceptives], their side effects, and then you can choose better what you* want. *They shouldn’t just give you Depo* after *having a baby you should be able to choose…*

Another woman in the same group discussion concurred with the need for comprehensive contraceptive counseling especially around possible side effects and a method tailored to women’s individual needs.

“*I do not feel happy – I am not enjoying the IUD because I don’t know what the consequences or disadvantages are as they [ nursing staff] won’t explain anything”. I have heard that a lot of women are not happy with the IUD.”*

Many women attributed unintended pregnancies to inadequate services received in family planning clinics including poor or absent provider-patient communication and counseling. Rude staff, long waiting times and often having to return on another day increased the risk of an unintended pregnancy, as explained by a female focus group participant.

“*You must come back and make another appointment to get contraception, they say I won’t help you today ...and you get busy, and that’s why most people end up being pregnant, it’s because they are rude, they can’t assist people in a good way*. *They don’t know that they are there for services to serve people*”*.*

### Partner relations/gender dynamics

Contraception and family planning were viewed by women in different ways, from being essential to preventing an unintended pregnancy, as a means to acquire greater reproductive autonomy, to notions of being a “good woman” or “mother”.

“*A good woman takes care of her body … I feel proud about myself because I use contraception and because I want to rear my children well”.*

Contraception was seen as an inevitable part of a woman’s reproductive role. A woman recounted:

*“We don’t use contraception because we love it, we use it because we just don’t want to get pregnant”.*

For some women, negotiating contraceptive use, especially male condoms, was difficult and challenged their reproductive autonomy*:*

“*I don’t forget it [ contraception]. And also I’m in a long-term relationship and as much as men have this connotation of they like skin-on-skin, I’m persistent. It’s my body, I’m the one who’s going to fall pregnant, not you, so please, respect that and if you can’t use a condom, then there’s no sex*”.

## Discussion

To our knowledge this is the first study in South Africa exploring women’s embodied experiences of contraception and family planning through body mapping techniques. Our study suggests that body mapping is an effective method to explore women’s embodied experiences of reproduction, conception and contraception. While some aspects including reproductive knowledge and beliefs have been explored elsewhere [12–16], this is the first time that the broader issues of women’s everyday life contexts and the influence of wider social networks on contraceptive decision making have been explored.

Numerous factors influenced women’s experiences related to contraceptive uptake, initiation and use. Physical, tactile and sensory attributes of contraceptive methods and knowledge gleaned from social and community networks all influenced contraceptive use. Notions of autonomy associated with contraceptive use was reported by some women and has been reported elsewhere in similar contexts [17]. Limited levels of knowledge around sexual and reproductive health, including contraceptive options and family planning methods, were evidenced by many women. Women shared their need and desire for reproductive health counseling, information sharing, especially with health care providers, and a person-centred approach to enable informed decision making around reproductive health needs. Person or client-centred contraceptive counseling is drawing increasing attention, but little is known outside of the United States about what women value in contraceptive counselling [18–21]. Whilst in South Africa, studies have shown how healthcare providers’ own moral values and attitudes influence their service provision of sexual and reproductive health services, including contraception and abortion [22–24], this study is the first to show the relationship between healthcare providers’ attitudes and the uptake and preference for contraceptive methods. Our study highlighted women’s needs for improved patient provider counseling and recognition of women’s own local knowledge and personal frameworks and how this influences contraceptive initiation, uptake and use.

Women had limited understanding and experience with the concept of family planning as it related to pregnancy spacing and how to translate pregnancy spacing and planning into their everyday lives. This is similar to findings reported elsewhere [18]. Family planning was linked to socioeconomic imperatives, yet women had little opportunity or ability to put these ideas into practice. Despite fluid sexual and partner relations, opportunities for contraceptive decision making and family planning were perceived as having empowering possibilities. The need for comprehensive person-centred counseling on how contraception works, and different method attributes especially around menstrual changes and body weight, underscored a health system that was not supporting these needs and highlighted the need for innovative ways to impart knowledge to all involved in contraceptive decision making.

Although verbal research methods have been privileged, our experience of the world is multisensory. Our study set out to uncover meanings not obtained through more conventional verbally-driven research methods by using embodied, participatory research methods to generate novel participant-centred findings [15]. A multisensory participatory approach has been undertaken recently in South Africa in a somewhat different context through exploring the multisensory components of antiretroviral medicine taking amongst HIV-positive adolescents [25].

Body mapping has been found to be an innovative and adaptable research methodology within the field of sexual and reproductive health, that holds many benefits for researchers and participants alike [10–12].. We found that body mapping was an acceptable and appropriate methodology to uncover perceptions, preferences and sensory experiences of contraception. The value of gaining multi-faceted and deeper insight into local knowledge on sexual and reproductive health as well as bodily experiences of contraception through body mapping techniques holds promise for developing contraceptive services that are more appropriate and effective for women and their partners. Similarly, an exploration of indigenous perceptions of reproductive anatomy and contraception in Timor- Leste through body mapping techniques demonstrated that body mapping was an effective method to traverse language, culture and local insights around contraception and reproductive health knowledge and beliefs [12].

## Limitations and challenges

This study has limitations. The research was conducted in both peri-urban and urban areas within the Western Cape Province, South Africa, and the findings may not be generalizable to other parts of the country, especially rural areas where access to health services might be more challenging.

Some caution and possible limitations of body mapping within the context of sexual and reproductive health research has been noted [10, 13]. The possibility of over-interpretation or misinterpretation of body maps by researchers has been raised as a point of caution [13]. To reduce this risk, we triangulated the visual data with workshop observations and data from the group and individual discussions. Sustaining 1–2 day body mapping workshops is time and resource-intensive and limits the number of study participants, though some have argued that body mapping is one of the least resource-intensive participatory methods in contrast to participatory visual methods such as photo voice and digital storytelling that requires funding for technology [26]. We overcame some of the time constraints related to 2-day body mapping workshops by modifying the body mapping workshops to one day, using smaller body maps to allow us to increase our overall number of study participants.

## Conclusions

Women’s embodied experiences of contraceptive methods and their day-to-day lived experiences of contraceptive use influenced their understandings and decision-making around contraceptive initiation, uptake and effective use. The ways in which contraceptive counseling and communication are traditionally and currently provided does not take cognizance of, or give time and space to, the broader social contexts influencing uptake or women’s limited knowledge of reproductive health and how contraception works. Person- centred counseling and dynamic communication strategies could help bridge the vast knowledge and communication gaps between users and providers of contraceptive and family planning services in South Africa. Body mapping techniques in an adapted form could possibly be used in adolescent education and communication strategies around sexual and reproductive health programmes.

This study highlighted the need to address communication and knowledge gaps around the female reproductive anatomy, different contraceptive methods, how contraception works to prevent a pregnancy underscored by the influence of wider social networks and local knowledge. Women, including younger women, identified sexual and reproductive health knowledge gaps themselves and identified these gaps as important factors that influenced uptake and effective contraceptive use. These knowledge gaps were overwhelmingly linked to poor or absent communication and counseling provided by the health care services.

## Data Availability

The datasets used and/or analyzed during the current study are available from the corresponding authors upon reasonable request.

## Abbreviations

FGD: Focus group discussion
IUD: Intrauterine device
ZAR: South African Rand
